# An Exploratory Study of the Role of Dietary Proteins in the Regulation of Intestinal Glucose Absorption

**DOI:** 10.3389/fnut.2021.769773

**Published:** 2022-01-19

**Authors:** Camille Dugardin, Léa Fleury, Véronique Touche, Farah Ahdach, Jean Lesage, Mathie Tenenbaum, Nadia Everaert, Olivier Briand, Sophie Lestavel, Rozenn Ravallec, Benoit Cudennec

**Affiliations:** ^1^Univ. Lille, Univ. Artois, Université de Liège, UMRT 1158 BioEcoAgro – Bénéfice santé d'hydrolysats de protéines et coproduits agro-alimentaires, Lille, France; ^2^Univ. Lille, Inserm, CHU Lille, Institut Pasteur de Lille, U1011- EGID, Lille, France; ^3^Univ. Lille, Inserm, CHU Lille, U1286 - INFINITE, Lille, France; ^4^Animal and Human Health Engineering, Department of Biosystems, Katholieke Universiteit Leuven, Heverlee, Belgium

**Keywords:** digested proteins, intestinal glucose absorption, SGLT1, GLUT2, glucose tolerance

## Abstract

Several studies have demonstrated that high protein diets improve glucose homeostasis. Nevertheless, the mechanisms underlying this effect remain elusive. This exploratory study aims to screen and compare the acute effects of dietary proteins from different sources on intestinal glucose absorption. Six dietary proteins from various sources were thus selected and digested thanks to the INFOGEST static gastrointestinal digestion protocol. The digested proteins were able to decrease intestinal glucose absorption *in vitro* and *ex vivo*. Moreover, acute ingestion of casein and fish gelatin led to improved glucose tolerance in Wistar rats without significant effect on insulin secretion. In parallel, GLUT2 mRNA expression in enterocytes was decreased following short-term incubation with some of the digested proteins. These results strengthen the evidence that digested protein-derived peptides and amino acids are key regulators of glucose homeostasis and highlight their role in intestinal glucose absorption.

## Introduction

According to UN figures, the world population is expected to reach 9.5 billion in 2050, corresponding to an increase of almost 30% in 50 years (United Nations, 2019 Revision of World Population Prospects). This growth implies an increased demand for food, particularly for proteins, due to socio-economic changes such as rising incomes, increased urbanization, and recognizing the role of proteins in a healthy diet (1). Simultaneously, obesity and type 2 diabetes mellitus (T2DM) are becoming the primary and widespread globally chronic health problems, leading to a dysregulation of the control of energy balance and glucose homeostasis. Metformin is generally the first medication prescribed to people with T2DM since it helps to lower blood glucose levels by reducing hepatic glucose production and release. However, it has recently been suggested that the intestine is also involved in the blood glucose–lowering effect of metformin *via* the acute inhibition of intestinal glucose transport (2). T2DM can also be treated by other antidiabetic drugs such as dipeptidyl peptidase-4 (DPP-IV) inhibitors, which extend the half-life of the native incretins, such as the glucagon-like peptide-1 (GLP-1) and the glucose-dependent insulinotropic polypeptide (GIP).

To limit the side effects of prolonged use of synthetic molecules for therapeutic purposes, natural compounds and their derivatives, such as functional foods or nutraceuticals, appear as safer alternatives. Dietary proteins from animal or plant origins exert, notably through the action of peptides generated during their gastrointestinal digestion, a wide range of nutritional and biological functions with beneficial effects on various pathological symptoms (3).

Dietary proteins are key regulators of glucose homeostasis and could therefore be developed in the context of obesity and T2DM. Indeed, several studies conducted in rodents and humans have shown that high protein diets improve glucose homeostasis (4, 5). Furthermore, proteins are involved in the regulation of energy metabolism by increasing energy expenditure, promoting satiety, and helping to maintain lean mass (6). High protein diets are beneficial to improve body composition by promoting fat loss while maintaining lean mass, which is remarkably interesting in the management of T2DM. Dietary proteins also play a critical role in the control of energy balance, *via* the induction of satiety effect and weight loss in both animals and humans (7) and the stimulation of appetite-regulating gut hormone secretion such as cholecystokinin (CCK), peptide YY, and the incretin GLP-1 (8). Likewise, it has recently been evidenced that peptides generated by bean protein hydrolysis induce a decreased glucose uptake in Caco-2 cells (9), suggesting that the detection of digested-derived proteins at the intestinal level could also modulate glucose transport from the intestinal lumen to blood circulation. This transport mainly involves the Na+/glucose cotransporter (SGLT1) at the apical side of enterocytes and glucose transporter type 2 (GLUT2) at the basolateral side of enterocytes (10).

This exploratory study aimed to screen and compare the acute effects of 6 dietary proteins from different sources, specifically on intestinal glucose absorption using *in vitro* and *ex vivo* models. Their effect on glucose tolerance was thereafter studied *in vivo* in rats. In parallel, the acute effect of digested proteins on SGLT1 and GLUT2 mRNA expression was investigated *in vitro* and *in vivo*.

## Materials and Methods

### Protein Samples

The following protein samples from diverse origins were purchased:

- Hemoglobin from bovine blood (ref H2625, Sigma-Aldrich, Saint-Quentin Fallavier, France);
- Ovalbumin from chicken egg white (ref A5503, Sigma-Aldrich);
- Casein from milk (ref PRODIET 85B, Ingredia, Arras, France);
- Pea proteins (ref NUTRALYS S85F, Roquette, Lestrem, France);
- High molecular weight dried fish gelatin (Keney & Ross, Port Saxon, Canada);
- The gluten from wheat (ref G5004, Sigma-Aldrich), which was tested only once digested for *in vitro* and *ex vivo* experiments because of its too weak solubility for *in vivo* manipulations.

Total nitrogen content was determined by the Kjeldahl method (11) and multiplied by the appropriate conversion factor to determine crude protein content (Table 1).

**Table 1:** Protein content (%) of the different protein samples determined by the kjeldahl method.

**Protein sample**	**% Protein content**
Hemoglobin	88.8
Ovalbumin	76.4
Casein	83.0
Pea proteins	65.6
Fish gelatin	87.6
Gluten	71.7

### Simulated Gastrointestinal Digestion

For *in vitro* and *ex vivo* experiments, protein samples were first digested *in vitro* according to the INFOGEST harmonized protocol (12) adapted for protein alone (13). Briefly, 2 g of protein sample (adjusted to % protein content) was solubilized in 8 ml of water and added to the reactor containing 8 ml simulated salivary fluid (SSF – Table 2) at pH 7.0, for 5 min. Then, 12 ml of simulated gastric fluid (SGF – Table 2) containing pepsin (ref P6887, 6,500 U/ml, Sigma-Aldrich) were added after saliva sampling (4 ml) and the pH solution was adjusted to 3.0 for 2 h. Finally, 20 ml of simulated intestinal fluid (SIF – Table 2) containing pancreatin (ref P1750, 45 U/ml, Sigma-Aldrich) were added to the batch after gastric sampling (4 ml), and the pH solution was adjusted to 7.0 for 2 h. Intestinal digest concentration reaches 31.25 mg/ml. After heating at 95°C for 10 min to assure enzyme denaturation without peptide damage, all samples were centrifuged at 13,400 g for 10 min at room temperature. Supernatants were collected and frozen at −20°C for further analysis. A blank SGID (without any protein sample but only with digestive enzymes) was also prepared in order to identify any activity due to enzyme autolysis in all further analyses.

**Table 2:** Chemical composition, protease concentration, and pH used for the different compartments of the *in vitro* simulated gastrointestinal digestion.

	**SSF**	**SGF**	**SIF**
Chemical composition	KCl (15.1 mM)	KCl (6.9 mM)	KCl (6.8 mM)
	KH_2_PO_4_ (3.7 mM)	KH_2_PO_4_ (0.9 mM)	KH_2_PO_4_ (0.8 mM)
	NaHCO_3_ (13.6 mM)	NaHCO_3_ (25 mM)	NaHCO_3_ (85 mM)
	–	NaCl (47.2 mM)	NaCl (38.4 mM)
	MgCl_2_ (0.15 mM)	MgCl_2_ (0.12 mM)	MgCl_2_ (0.33 mM)
	NH_4_HCO_3_ (0.06 mM)	NH_4_HCO_3_ (0.5 mM)	–
	CaCl_2_ (H_2_O)_2_ (1.5 mM)	CaCl_2_ (H_2_O)_2_ (0.15 mM)	CaCl_2_ (H_2_O)_2_ (0.6 mM)
Proteases		Pepsin 1:35 (w:w)	Pancreatin 1:2.75 (w:w)
pH	7.0	3.0	7.0

### Cell Culture

#### Caco-2/TC7 Cells

Caco-2/TC7 cells (from Pr. Chambaz, Centre de recherche des Cordeliers, Paris, France) were routinely grown in 25 cm^2^ culture flasks (ref Biolite, Thermo Fisher Scientific, Waltham, USA), under a humidified atmosphere containing 10% CO_2_, at 37°C, in Dulbecco's modified essential medium (DMEM) containing 25 mM glucose and L-glutamine (Gibco, Dun Laoghaire, Ireland) supplemented with penicillin/streptomycin (100 U/ml and 100 μg/ml, respectively), 1% non-essential amino acids, and 20% heat-inactivated fetal calf serum.

#### Caco-2 Cells

Caco-2 cells (Sigma–Aldrich) were routinely grown in 75 cm^2^ culture flasks (Sarstedt), under a humidified atmosphere containing 5% CO_2_, at 37°C, in DMEM (PAN Biotech, Aidenbach, Germany) supplemented with penicillin/streptomycin (100 U/ml), 2 mM L-glutamine, and 10% heat-inactivated fetal bovine serum.

#### HT29-MTX

Mucus-secreting HT29-MTX cells (Sigma–Aldrich) were routinely grown in 75 cm^2^ culture flasks (Sarstedt, Nümbrecht, Germany), under a humidified atmosphere containing 5% CO_2_, at 37°C, in DMEM (PAN Biotech) supplemented with penicillin/streptomycin (100 U/mL), 2 mM L-glutamine, and 10% heat-inactivated fetal bovine serum.

### *In vitro* Glucose Uptake

Caco-2/TC7 cells were seeded at a density of 250,000 cells per 4.2 cm^2^ transwells (microporus PET membrane, 3 μm pore size, Falcon, Corning, New York, USA) and grown in supplemented DMEM to reach confluence after 1 week. Cells were further cultivated for 2 weeks with serum-free DMEM in the upper compartment and supplemented medium in the lower compartment. For uptake experiments, after 1-h of apical pre-incubation with 5 mg/ml digested proteins diluted in non-supplemented DMEM, cells were apically exposed for 40 min to the same digested protein-containing medium supplemented with 2 mM α-methyl-D-glucopyranoside (AMG) and 0.2 μCi/ml ^14^C-α-methyl-D-glucopyranoside (PerkinElmer, Waltham, USA). Uptake was stopped by washing the cells with cold PBS containing 0.5 mM phlorizin. Cells were solubilized in Solvable (PerkinElmer, Waltham, USA). Radioactivity in the cellular compartment was measured using a scintillation counter (TopCount NTX, PerkinElmer). Protein concentrations of lysates were measured using Pierce BCA protein assay (Thermo Fisher Scientific).

### *Ex vivo* Glucose Absorption

The experiments were performed using jejunal sacs from 16 h-fasted adult Wistar rats. The proximal jejunum was dissected and rinsed in a cold saline solution. Jejunal sacs (1 cm length) were prepared for ^3^H-D-[1-14C] glucose (49.5 mCi/mmol) transport as previously described (14). Jejunal sacs were filled with 0.3 ml of Krebs–Ringer Modified Buffer (KRB) containing digested proteins at 31.25 mg/ml and 0.02 Ci/ml of the isotopic tracer ^3^H-D-[1-^14^C] glucose supplemented by glucose to obtain a final concentration of 30 mM. Transmural glucose passage was quantified by measuring ^3^H-D-[1-^14^C] glucose release in the incubation medium for 20 min. Transport of the isotopic tracer was normalized to the weight (mg) of the jejunum segment. The area under the curve (AUC) was then calculated.

### Animal Care

Experiments were performed on 12-week-old Wistar male rats purchased from Envigo (Gannat, France) according to the European Union guidelines for the use of laboratory animals and in compliance with the French ethical guidelines for studies on experimental animals (Animal house agreement no. 5900912, Authorization for Animal Experimentation no. 20992-201906031147321 v3, project approval by our local ethical committee no. CEEA75). Rats were housed in individual cages, in a temperature and humidity controlled room (22 ± 2°C), under a 12 h light–dark cycle, and fed *ad libitum* with a standard rodent diet (ref 3430PMS10, Serlab, Montataire, France). After 1 week of acclimatization, they were trained for intragastric administration with water for 3 days. At the end of the 1st week, rats were all weighed and distributed in 8 groups of homogeneous weight (*n* = 8).

### Oral Glucose Tolerance Test

After overnight fasting, rats were gavaged with 1 g.kg^−1^ bodyweight of protein samples (1.5 mL of proteins at 233 mg/mL) or water (control group) using a rigid curved gavage needle, 30 min before glucose load (2.5 g/kg bodyweight). Blood samples were collected *via* the tail vein before (T0) and 15, 30, 60, 90, and 120 min after the oral glucose load. Blood was collected in tubes containing EDTA and plasma was prepared by centrifugation. Plasma glucose and insulin were, respectively, determined using a glucometer (Accu-Chek, Roche, Bâle, Switzerland) and ELISA (ref EZRMI-13K, Merck Millipore, Burlington, USA) and incremental area under the curve (iAUC) calculated for both.

### Total RNA Extraction, cDNA Synthesis, and RT-qPCR Analysis

#### Caco-2/TC7 Cells

Caco-2/TC7 cells were seeded in 12-well plates at a density of 60,000 cells per cm^2^ in a volume of 1,000 μl supplemented DMEM and grown at 37°C, 10% CO_2_ for 10 days. On the day of the experiment, cells were washed with PBS and 1,000 μl of each digested protein was added at 5 mg/ml (diluted in non-supplemented DMEM). At the end of incubation time (4 h), the supernatant was removed and RNA was extracted using TRIzol reagent (Life technologies, Carlsbad, USA). RNA concentration and purity were assessed using the Nanodrop ND-1000 (Thermo Fisher Scientific). Reverse transcription was performed using High-Capacity cDNA Reverse Transcription Kit (Applied Biosystems, Waltham, USA) and mRNA levels of interest were determined by qPCR on an MX3005 device (Agilent Technologies, Santa Clara, USA) using the Takyon Low Rox Probe MasterMix dTTP blue (Eurogentec, Seraing, Belgium) and specific primers (Table 3).

**Table 3:** Primers used for qPCR experiments.

**Gene**	**Model**	**Forward primer**	**Reverse primer**
SGLT1	Caco-2/TC7 cells	TGGCAATCACTGCCCTTTAC	CAGGATTAAAGACCCCACCA
	Caco-2/HT29-MTX	CCGGATGTGCATCTCTACCG	TGGTCTCCTTAGGGCCTTCTT
	Rat jejunum	TTCGAATGGAACGCCTTGGT	GATCTGGATCCGCTTGCCTC
GLUT2	Caco-2/TC7 cells	GGAGTTGGCGCTGTAAACAT	CAAACATCCCACTCATTCCA
	Caco-2/HT29-MTX	CGGTGAAATTGCTCCAACCG	GCAGGATGTGCCACAGATCA
	Rat jejunum	GCAACATGTCAGAAGACAAGATCAC	GAGGTGCATTGATCACACCGA
TBP	Caco-2/TC7 cells	GGAGAGTTCTGGGATTGTACCGC	ATATTCGGCGTTTCGGGCAC
HPRT1	Caco-2/HT29-MTX	GCCCTGGCGTCGTGATTAGT	GCAAGACGTTCAGTCCTGTCC
	Rat jejunum	TGCTTTCCTTGGTCAAGCAGT	AATCCAACAAAGTCTGGCCTGT

#### Caco-2/HT29-MTX Co-culture

Caco-2/HT29-MTX cells were seeded at a ratio of 90/10 in 24-well plates at a density of 20,000 cells per cm^2^ in a volume of 500 μl supplemented DMEM and grown at 37°C, 5% CO_2_ for 15 days. On the day of the experiment, cells were washed with PBS and 500 μl of each digested protein was added at 5 mg/ml (diluted in non-supplemented DMEM). At the end of incubation time (2 or 4 h), the supernatant was removed and RNA was extracted using the modified NucleoZOL (Macherey-Nagel, Düren, Germany) protocol. RNA concentration and purity were assessed using the Nanodrop lite. Reverse transcription was performed using RevertAid H Minus First Strand cDNA Synthesis Kit (Thermo Fisher Scientific) and mRNA levels were determined by qPCR on a CFX Connect Real-Time PCR detection system (BioRad, Hercules, USA) using the Takyon No Rox SYBR MasterMix dTTP Blue (Eurogentec) and specific primers (Table 3).

#### Rat Jejunum

After 14 h-fasting, rats were gavaged with 1 g/kg bodyweight of protein samples or water (control group) and sacrificed 30 min later (*n* = 7/8). Scraped mucosa from the jejunum was collected. Rat intestinal jejunum RNA was extracted using the modified NucleoZOL (Macherey–Nagel) protocol. RNA concentration and purity were assessed using the Nanodrop lite. Reverse transcription was performed using RevertAid H Minus First Strand cDNA Synthesis Kit (Thermo Fischer Scientific) and mRNA levels were determined by qPCR on a CFX Connect Real-Time PCR detection system (Biorad) using the Takyon No Rox SYBR MasterMix dTTP Blue (Eurogentec) and specific primers (Table 2).

### Statistical Analysis

All data were expressed as mean values with their standard deviations. GraphPad Prism was used to calculate AUC and to carry out statistical analysis. All significances *vs*. the control group were measured using either one-way ANOVA followed by Dunnett's test or two-way ANOVA followed by Dunnett's test.

## Results

### Decrease of Intestinal Glucose Absorption in Response to Digested Dietary Proteins

**Figure 1:**
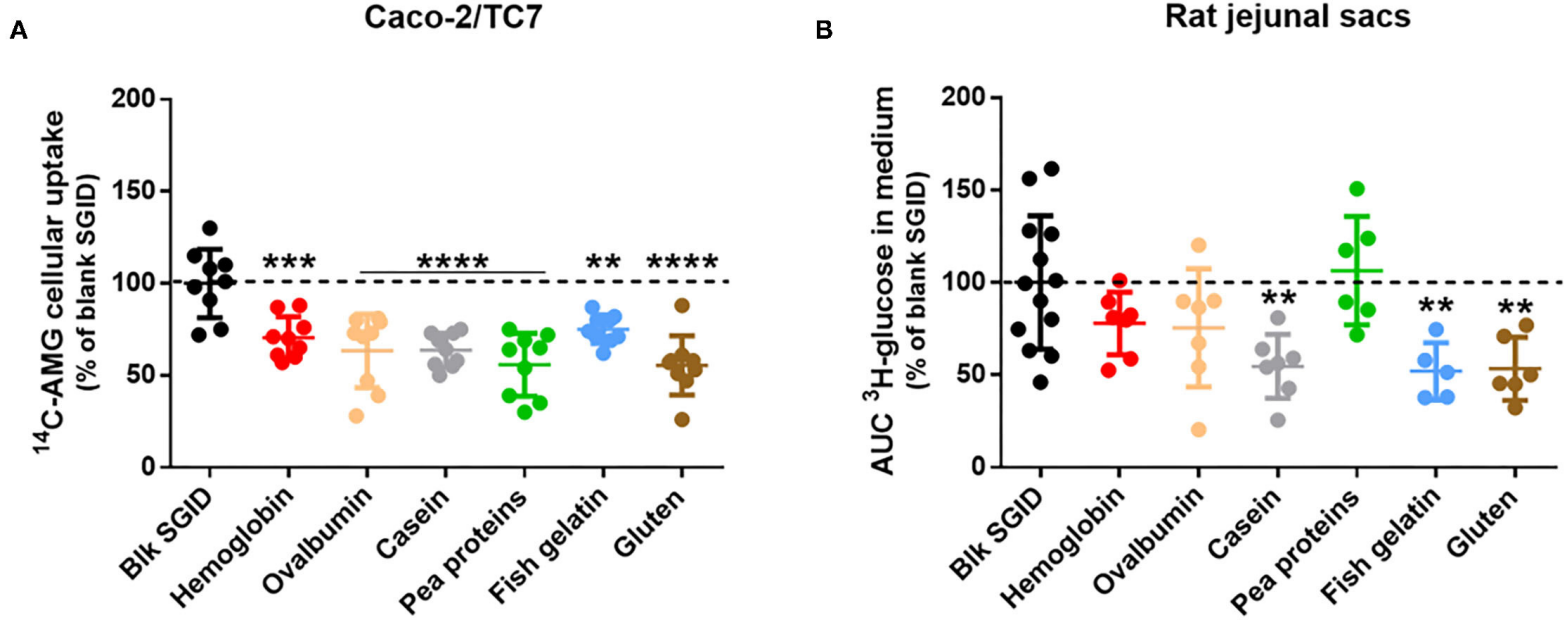
Some digested dietary proteins decrease intestinal glucose absorption *in vitro* and *ex vivo*. **(A)** After 1-h apical pre-incubation with 5 mg/ml digested proteins, Caco-2/TC7 cells differentiated on transwells were apically exposed for 40 min to the same digested proteins supplemented with ^14^C-α-methyl-D-glucopyranoside (AMG). Glucose uptake was then quantified by measuring radioactivity in the cellular compartment. Results are expressed as % of control (blk SGID) – mean ± SD (**, *p* < 0.01; ***, *p* < 0.001; and ****, *p* < 0.0001 compared to control – one-way ANOVA followed by Dunnett's test). **(B)** Rat jejunal sacs were filled with 31.25 mg/mL digested proteins (hemoglobin, ovalbumin, casein, pea proteins, fish gelatin, and gluten) and ^3^H-glucose. Glucose transport was quantified by measuring radioactivity in the incubation medium for 20 min and AUC was then calculated. Results are expressed as % of control (blk SGID) – mean ± SD (**, *p* < 0.01 compared to control – one-way ANOVA followed by Dunnett's test).

The acute effect of different digested dietary proteins (hemoglobin, ovalbumin, casein, pea proteins, fish gelatin, and gluten) on intestinal glucose absorption was evaluated using *in vitro* and *ex vivo* models. First, gastrointestinal digestion of the dietary proteins was simulated following the recommendation of the consensual INFOGEST *in vitro* protocol (12). A blank SGID was also performed as a control. Differentiated Caco-2/TC7 cells on transwells were then pre-incubated for 40 min with the different digested proteins before being apically exposed to an SGLT1-specific glucose analog (AMG) to evaluate enterocyte glucose uptake. All digested proteins significantly decreased ^14^C-AMG uptake at the apical side of human enterocytes compared to the blank SGID (Figure 1A). To strengthen these *in vitro* results, rat jejunal sacs were filled with the different digested dietary proteins, and glucose transport from the intestinal lumen to the extracellular medium was assessed using a radioactive tracer. Incubation of rat jejunal sacs with digested casein, fish gelatin, and gluten significantly decreased intestinal glucose transport compared to the control blank SGID (Figure 1B).

### Acute Effects of Proteins on Glucose Tolerance in Rats

**Figure 2:**
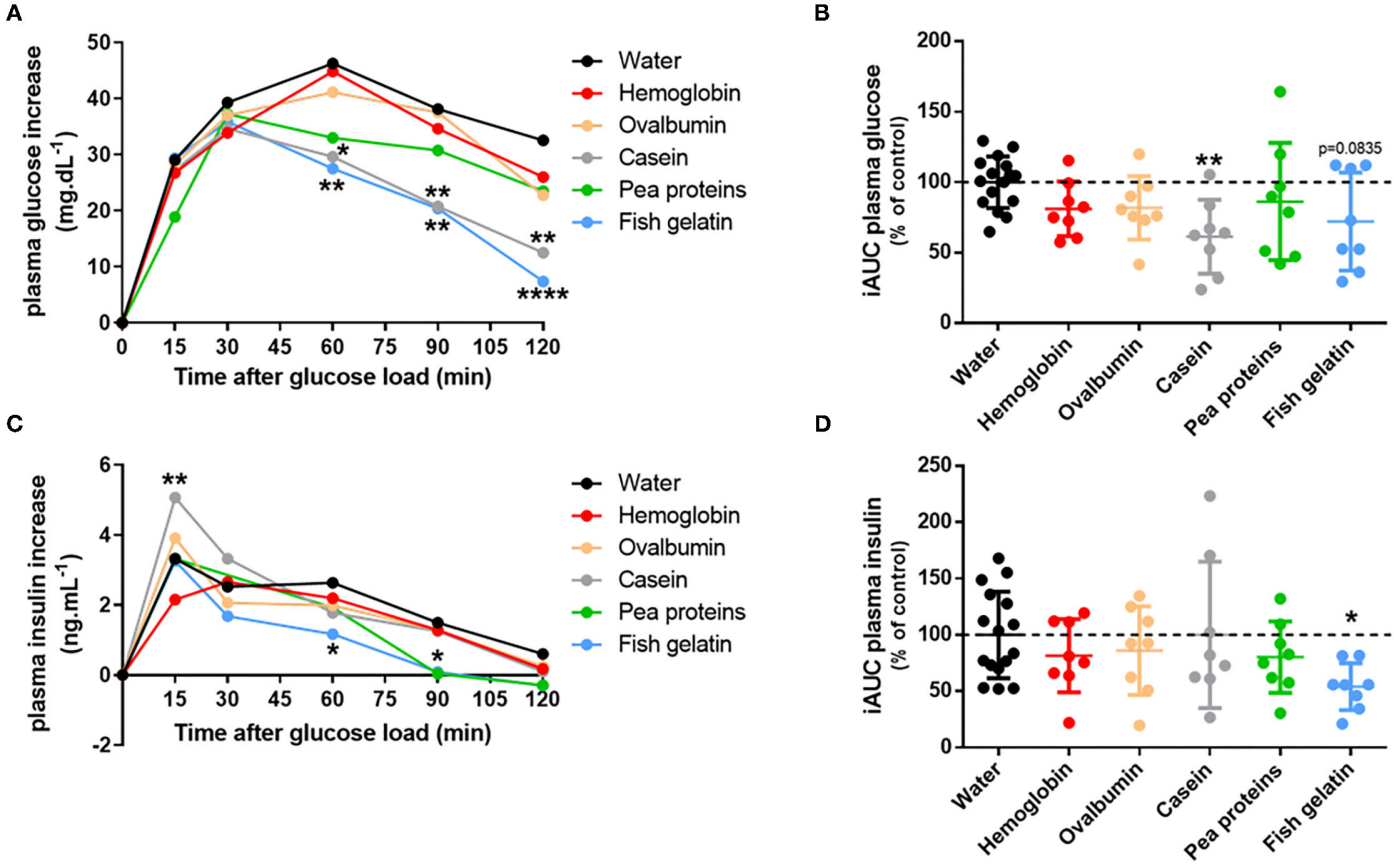
Acute ingestion of casein and fish gelatin improves glucose tolerance in rats. Overnight fasted rats were first given either water or protein (hemoglobin, ovalbumin, casein, pea proteins, or fish gelatin) at a dose of 1 g/kg bodyweight by oral gavage (*n* = 8); 30 min later, glucose was orally administered at a dose of 2.5 g/kg bodyweight to start OGTT. **(A)** Mean plasma glucose increase during OGTT expressed in mg/dL (*, *p* < 0.05; **, *p* < 0.01; and ****, *p* < 0.0001 compared to control (water) – two-way ANOVA followed by Dunnett's test) and **(B)** the corresponding iAUC values expressed as % of control (water) – mean ± SD (**, *p* < 0.01 compared to control – one-way ANOVA followed by Dunnett's test). **(C)** Mean plasma insulin increase during OGTT expressed in ng/mL (*, *p* < 0.05 and **, *p* < 0.01 compared to control (water) – two-way ANOVA followed by Dunnett's test) and **(D)** the corresponding iAUC values expressed as % of control (water) – mean ± SD (*, *p* < 0.05 compared to control – one-way ANOVA followed by Dunnett's test).

Consequently, the acute effect of the different proteins on glucose tolerance was evaluated. To do that, overnight fasted rats were gavaged with proteins (hemoglobin, ovalbumin, casein, pea proteins, and fish gelatin) and received glucose by gavage 30 min later to perform an oral glucose tolerance test (OGTT). There was no statistical difference in baseline blood glucose (time 0) between the treatments. Acute ingestion of casein and fish gelatin improved glucose tolerance, as evidenced by the decreased blood glucose concentrations during OGTT (Figure 2A). In the same way, the calculated incremental area under the glucose curve (iAUC) was decreased in casein (*p* < 0.01) and nearly for fish gelatin (*p* = 0.0835) ingested groups as compared to water controls (Figure 2B). Plasma insulin levels were also determined during OGTT (Figure 2C) and iAUC calculated for each rat (Figure 2D). Acute ingestion of casein induced a significantly higher peak at 15 min of insulin secretion than the control group, whereas acute ingestion of fish gelatin and pea proteins induced significantly lower insulin secretion, respectively, after 60 and 90 min (Figure 2C). Regarding iAUC, despite a lowering trend, no significant effect was highlighted, except for the fish gelatin group whose iAUC is lower than of the water group (*p* < 0.05) (Figure 2D).

### Regulation of GLUT2 mRNA Expression in Response to Digested Dietary Proteins

**Figure 3:**
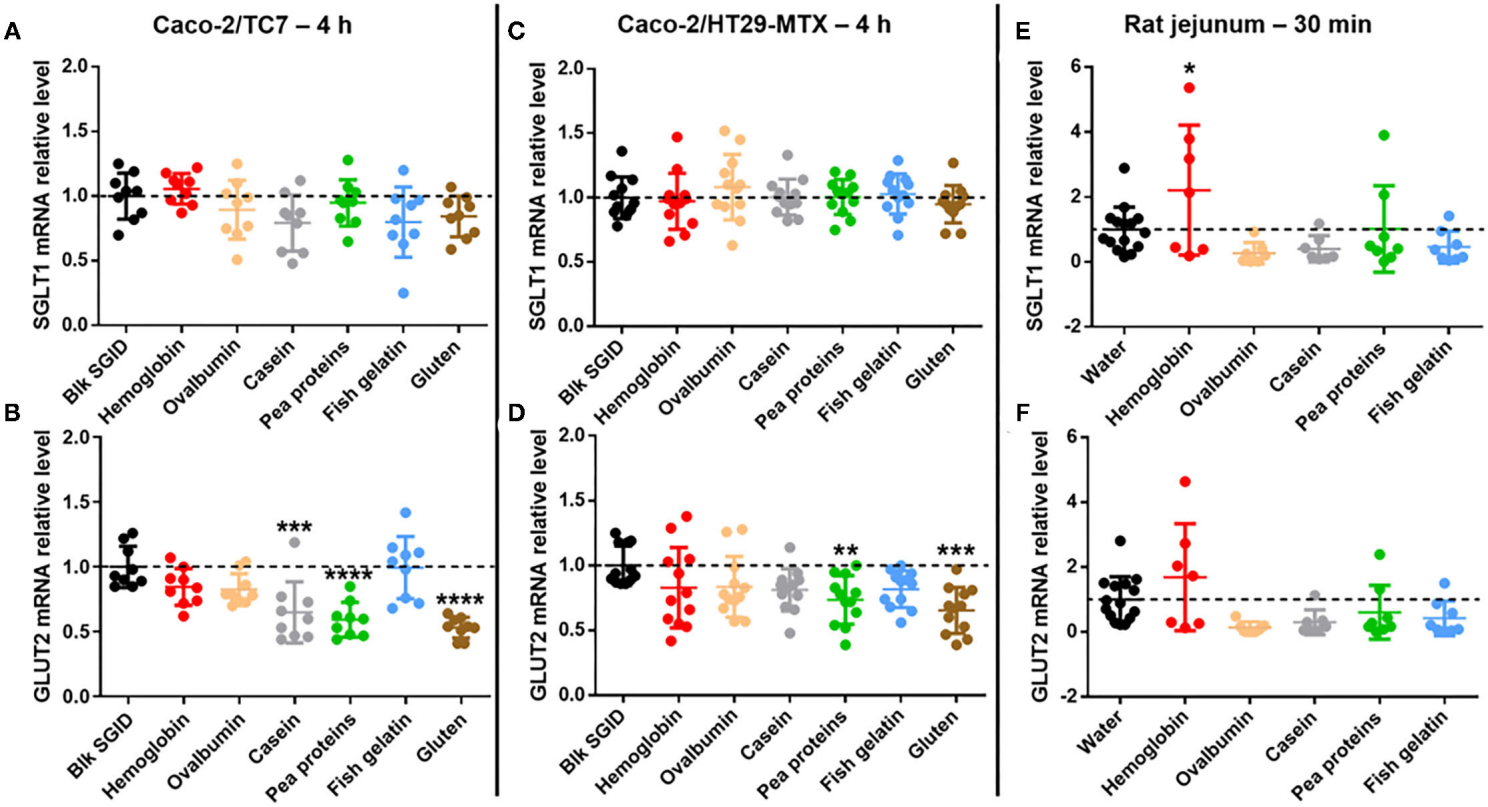
Some digested dietary proteins decrease GLUT2 mRNA expression *in vitro*. **(A,B)** SGLT1 **(A)** and GLUT2 **(B)** mRNA relative levels normalized to TBP in Caco-2/TC7 cells incubated for 4 h with 5 mg/mL digested proteins (hemoglobin, ovalbumin, casein, pea proteins, fish gelatin, and gluten). Control condition (Blk SGID) is set at 1. Mean ± SD (***, *p* < 0.001 and ****, *p* < 0.0001 compared to control – one-way ANOVA followed by Dunnett's test). **(C,D)** SGLT1 **(C)** and GLUT2 **(D)** mRNA relative levels normalized to HPRT1 in Caco-2/HT29-MTX co-culture incubated for 4 h with 5 mg/ml digested proteins (hemoglobin, ovalbumin, casein, pea proteins, fish gelatin, and gluten). Control condition (Blk SGID) is set at 1. Mean ± SD (**, *p* < 0.01 and ***, *p* < 0.001 compared to control – one-way ANOVA followed by Dunnett's test). **(E,F)** SGLT1 **(E)** and GLUT2 **(F)** mRNA relative levels normalized to HPRT1 in rat jejunal scraped mucosa, 30 min after gavage with 1 g/kg of bodyweight of dietary proteins (hemoglobin, ovalbumin, casein, pea proteins, and fish gelatin). Control condition (water) is set at 1. Mean ± SD (*, *p* < 0.05 compared to control – one-way ANOVA followed by Dunnett's test).

In parallel and to go further in this exploratory study, the acute effect of the different digested dietary proteins on the expression of the two main intestinal glucose transporters was investigated using different models. First, SGLT1 and GLUT2 mRNA expression was studied in Caco-2/TC7 cells (Figures 3A,B) but also in a Caco-2/HT29-MTX co-culture (Figures 3C,D) after a 4 h incubation with 5 mg/ml digested proteins (hemoglobin, ovalbumin, casein, pea proteins, fish gelatin, and gluten). Although short-term incubation with all digested proteins did not affect SGLT1 expression in Caco-2/TC7 cells (Figure 3A), digested casein, pea proteins, and gluten significantly decreased GLUT2 mRNA levels compared to the control blank SGID (Figure 3B). In Caco-2/HT29-MTX co-culture, no effect of digested proteins was observed on SGLT1 expression (Figure 3C). However, all digested proteins tend to decrease GLUT2 mRNA levels compared to the control blank SGID, with significant effect for digested pea proteins and gluten (digested casein, *p* = 0.1141) (Figure 3D). Overnight fasted rat jejunum was collected 30 min after protein gavage (hemoglobin, ovalbumin, casein, pea proteins, and fish gelatin) and jejunal mucosa was scraped to analyze SGLT1 and GLUT2 mRNA expression. Even though there was a decreasing tendency except for hemoglobin, no significant effect of dietary proteins on SGLT1 (Figure 3E) and GLUT2 mRNA (Figure 3F) levels was highlighted *in vivo* compared to water controls.

## Discussion

While the world population increases, it is necessary to promote a sustainable diet that can meet nutritional recommendations and economic and ecological concerns (15). In this context, government and public health authorities recommend integrating more plant proteins into the diet and reducing animal protein consumption [PNNS, 2020 (16)]. The protein source is concomitantly more and more crucial in terms of impact on health and ecology highlighting the urgent need for better characterization and rational use of dietary proteins. In this context, this study was designed to explore the acute effect of animal and vegetal proteins from diverse origins on glucose homeostasis *in vitro* and *in vivo*.

In this exploratory study, we have demonstrated, using Caco-2/TC7 cells differentiated on transwells, that *in vitro* digested dietary proteins, coming from different sources, could acutely decrease intestinal glucose uptake at the apical membrane of human enterocytes. Then, we evidenced for the first time that some of those digested proteins could also acutely decrease intestinal glucose transport *ex vivo*, using rat jejunal sacs. This decrease was significant for digested casein, fish gelatin, and gluten. Whereas, rat jejunal sacs permit the measurement of the whole intestinal glucose transport across the intestinal mucosa, the Caco-2/TC7 cell model allows studying specifically SGLT1-specific glucose uptake at the apical side of enterocytes thanks to the use of AMG. Another experiment was performed using radioactive 2-deoxyglucose (2DG) in Caco-2/TC7 cells instead of AMG, and similar results were obtained: all digested dietary proteins coming from different sources could acutely decrease intestinal 2DG uptake at the apical membrane of enterocytes (Supplementary Figure 1A) and consequently its transport through the basolateral membrane (Supplementary Figure 1B).

After the results were obtained, dietary proteins were tested for their ability to improve glucose tolerance in rats. In humans, some proteins and amino acids have been shown to improve glucose homeostasis by stimulating insulin and incretin hormone secretion (17, 18). In T2DB, it has also been highlighted that dietary proteins from different origins (meat, milk) could acutely improve glucose tolerance (19, 20). This effect was notably observed for animal-sourced proteins thanks to their high content of branched-chain amino acids. Among animal proteins, whey and cod proteins have shown remarkable results in improving acute glucose response. Indeed, a sufficient dose (≥ 0.8 g/kg/day) of these proteins associated with reduced carbohydrate intake diminished the glycemic response by increasing insulin secretion and sensitivity in healthy adults (21–23). However, the long-term effect of high animal protein intake on glucose homeostasis is not yet clearly demonstrated, but it could lead to insulin resistance (17). Consumption of a high animal protein diet is associated with increased cardiovascular risk and diabetes incidence (24). This reverse effect may be partly due to a rise in the consumption of animal products related to higher consumption of saturated fatty acids and cholesterol. The impacts of the quantity and the quality of proteins for maintaining or improving blood glucose are not yet established. Proteins that best improve glucose tolerance by stimulating insulin are animal proteins and, more precisely, casein (18, 25). In our study, short-term fish gelatin also improved blood glucose but without increasing insulin level. This result suggested that fish gelatin-induced lowering effect on blood glucose during the OGTT cannot be explained by an increase of plasma insulin levels and thus involves some alternative mechanisms. In a study comparing cod, soy, and casein proteins on glucose tolerance in rats, the same differences between cod and casein proteins on glucose and insulin levels were found (26). Fish gelatin and casein seem to act by different mechanisms to regulate blood glucose. Ingestion of casein improved blood glucose levels thanks to an increase in insulin secretion after 15 min, contrary to fish gelatin, which improved blood glucose levels after 60 min without involving insulin secretion. In a study, it was proposed that fish proteins could improve insulin sensitivity (27).

In parallel, we have shown that short-term incubation (4 h) with some digested dietary proteins led to decreased GLUT2 mRNA level without affecting SGLT1 mRNA expression in Caco-2/TC7 cells. We have reproduced and confirmed these results after 4 h but also 2 h-incubation with digested dietary proteins (Supplementary Figure 2) in a Caco-2/HT29-MTX co-culture model, which includes mucus-producing cells (28, 29). SGLT1 and GLUT2 mRNA levels were also evaluated in rat jejunum 30 min after protein gavage, but only a decreasing tendency could be highlighted compared to water controls. Whereas, the primary route for intestinal glucose transport from the intestinal lumen to blood circulation involves SGLT1 at the apical side of enterocytes and GLUT2 at the basolateral side of enterocytes, it was proposed that, in response to high glucose luminal concentrations (≥ 25 mM), a pool of endosomal GLUT2 is transiently translocated to the apical membrane to increase intestinal glucose uptake (30). While SGLT1 and GLUT2 mRNA level analysis in response to digested dietary proteins in Caco-2/TC7 cells and Caco-2/HT29-MTX co-culture were performed in high glucose conditions (25 mM), it was not the case for rat jejunum since animals fasted overnight before the experiment. This suggests that when glucose concentration is high, digested protein sensing at the enterocyte level could acutely regulate and decrease GLUT2 expression. The peptide-lowering effect on intestinal glucose uptake has already been suggested (9). Indeed, in this study, peptides derived from black bean hydrolysis decreased glucose uptake and GLUT2 expression (mRNA and protein) as well as its location at the apical enterocyte membrane in Caco-2 cells after 30 min incubation. Other studies are needed to better understand SGLT1 and GLUT2 roles in that process and to evaluate their protein level and localization at the enterocyte membrane in response to acute protein intake. Some other pathways are still to be explored to explain the effect of dietary proteins on intestinal glucose absorption such as GLUT2-independent mechanisms (31). Moreover, the results of the present study strongly corroborate the existence of intestinal nutrient sensing to modulate glucose homeostasis (32) and suggest potential crosstalk between glucose sensing and peptide sensing at the enterocyte level. Peptide transporter 1 (PepT1) is a high-capacity, low-affinity di- and tripeptide transporter expressed at the brush border membrane. A recent study has already demonstrated the role of PepT1 in the detection of casein hydrolysate in the rat's upper small intestine to regulate the production of glucose (33). It has been also shown that PepT1 and SGLT1 are both regulated by the sodium–hydrogen exchanger 3 (NHE3) located at the brush-border membrane. Indeed, whereas NHE3 post-transcriptionally increases the transport activity of PepT1 (34), the inhibition of NHE3 expression increases the expression and activity of SGLT1 in non-transformed small intestinal epithelial IEC-18 cells and *vice versa* (35), suggesting an opposite regulation of peptide and glucose intestinal uptake. Furthermore, whereas high glucose concentration (75 mM) in perfused rat small intestine leads to increased GLUT2 expression at the apical membrane of enterocytes, it decreases PepT1 expression (36). Our hypothesis is that peptide detection at the apical side of enterocytes could reciprocally decrease GLUT2 expression. Our results even suggest that the concomitant presence of digested proteins and high glucose concentration in the intestinal lumen could favor peptide sensing over glucose sensing. Other studies are needed to assess PepT1 involvement in digested protein-induced intestinal glucose uptake regulation.

During the digestion of food proteins, the action of digestive proteases in the different compartments of the gastrointestinal tract generates peptides of varying size, sequence, and structure, which can exert numerous biological activities (37). However, studies comparing the effect of different protein sources on their bioactivities are limited (38–40). The aim of the present work was thus to compare the acute effect of animal and vegetal proteins from diverse origins on glucose homeostasis. Experiments were performed on *in vitro* and *ex vivo* models, using proteins that were first digested thanks to the consensual INFOGEST *in vitro* static protocol (12), but also *in vivo* in rats, using non-digested proteins. Although this static digestion method is simple to use and well-recognized, it does not take into account digestion kinetics nor the influence of microbiota during digestion. Moreover, it is difficult to predict *in vivo* outcomes from *in vitro* studies since they lack many other glucose-regulating factors. Thus, casein and fish gelatin have shown the most promising effect on intestinal glucose transport in rat jejunum and glucose tolerance in rats. Gluten also decreased intestinal glucose transport across the rat intestine, but its effect on glucose tolerance could not be tested because of its too weak solubility for oral administration. However, all proteins tested effectively decreased glucose uptake in Caco-2/TC7 cells. Some of them also reduced GLUT2 mRNA expression in enterocytes, the best ones being pea proteins and gluten. The ability of several plant-based proteins (among which pea proteins) to modulate glucose homeostasis has already been highlighted *in vitro* through GLP-1 secretion induction and DPP-IV activity inhibition (40). Our study provides evidence that plant-based proteins could improve glucose homeostasis through the reduction of intestinal glucose uptake.

## Conclusions

**Figure 4:**
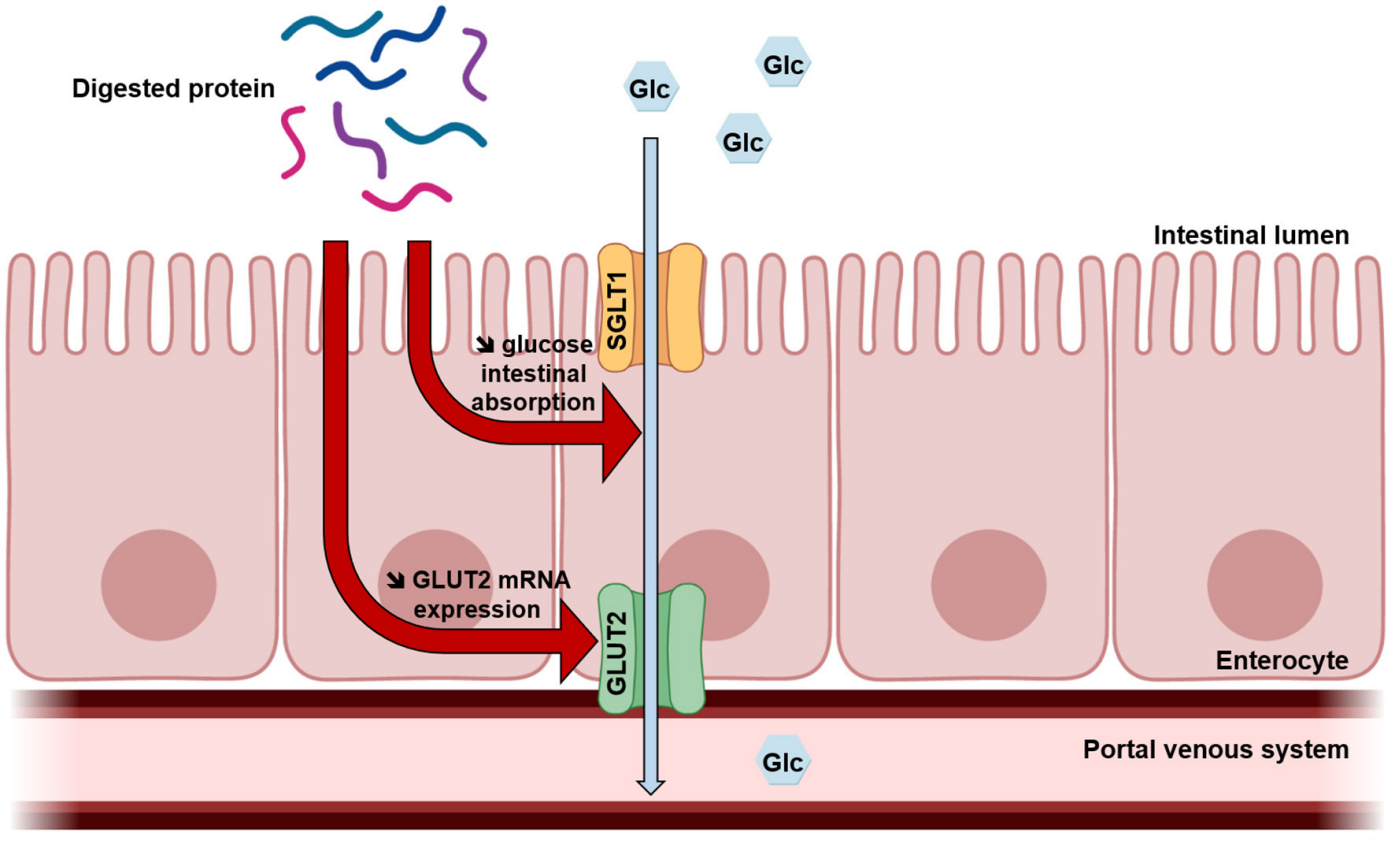
Proposed mechanism of digested protein effects on intestinal glucose absorption. Once digested, dietary proteins decrease intestinal glucose absorption and GLUT2 mRNA expression in enterocytes.

This exploratory study suggests for the first time that acute protein intake could improve glucose tolerance partly due to a lowering effect on intestinal glucose uptake (Figure 4). It is recognized that SGLT1-mediated glucose intestinal absorption is responsible for the rapid postprandial increase in blood glucose levels observed in obesity and T2DM (10). Moreover, some studies demonstrate that in this context, insulin resistance leads to increased intestinal glucose uptake, also due to the loss of GLUT2 trafficking control leading to permanent GLUT2 localization at the apical membrane (41, 42). The results of the present study highlight dietary proteins as effective regulators of intestinal glucose uptake, which could thus be developed to prevent obesity and treat T2DM in a context of protein resource rational use. Other studies are needed to investigate molecular mechanisms and better understand this regulation.

## Data Availability Statement

The raw data supporting the conclusions of this article will be made available by the authors, without undue reservation.

## Ethics Statement

The animal study was reviewed and approved by European Union guidelines for the use of laboratory animals and in compliance with the French Ethical guidelines for studies on experimental animal (Animal house agreement no. 5900912, Authorization for Animal Experimentation no. 20992-201906031147321 v3, and project approval by our Local Ethical Committee no. CEEA75).

## Author Contributions

CD, LF, VT, FA, JL, MT, RR, and BC performed the experiments. CD, LF, VT, and BC analyzed the data. CD, LF, and BC wrote the manuscript. VT, JL, OB, NE, SL, and RR contributed to the critical review of the manuscript. All authors approved the final manuscript for submission.

## Funding

This work has been carried out in the framework of ALIBIOTECH project which was financed by the European Union, French State, and the French Region of Hauts-de-France.

## Conflict of Interest

The authors declare that the research was conducted in the absence of any commercial or financial relationships that could be construed as a potential conflict of interest.

## Publisher's Note

All claims expressed in this article are solely those of the authors and do not necessarily represent those of their affiliated organizations, or those of the publisher, the editors and the reviewers. Any product that may be evaluated in this article, or claim that may be made by its manufacturer, is not guaranteed or endorsed by the publisher.

## Supplementary Material

Supplementary Figure 1 Some digested dietary proteins decrease 2-DG intestinal transport in Caco-2/TC7 cells. After 1 h apical pre-incubation with 5 mg/mL digested proteins, Caco-2/TC7 cells differentiated on transwells were apically exposed for 8 min to the same digested proteins supplemented with ^14^C-2-deoxyglucose (2DG). Glucose uptake and absorption were then quantified by measuring radioactivity, respectively, in the cellular **(A)** and basolateral **(B)** compartments. Results are expressed as % of control (blk SGID) – mean ± SD (^**^, *p* < 0.01; ^***^, *p* < 0.001; and ^****^, *p* < 0.0001 compared to control – one-way ANOVA followed by Dunnett's test).

Supplementary Figure 2 Some digested dietary proteins decrease GLUT2 mRNA expression after 2 h incubation in Caco-2/HT29-MTX co-culture. SGLT1 **(A)** and GLUT2 **(B)** mRNA relative levels normalized to HPRT1 in Caco-2/HT29-MTX co-culture incubated for 2 h with 5 mg/ml digested proteins (hemoglobin, ovalbumin, casein, pea proteins, fish gelatin, and gluten). Control condition (Blk SGID) is set at 1. Mean ± SD (^*^, *p* < 0.05 and ^**^, *p* < 0.01 compared to control – one-way ANOVA followed by Dunnett's test).
