# Supplementary figures and images for: An Exploratory Study of the Role of Dietary Proteins in the Regulation of Intestinal Glucose Absorption
Source: Front Nutr. 2022 Jan 19;8:769773. doi: 10.3389/fnut.2021.769773 (PMC8808719; doi:10.3389/fnut.2021.769773)

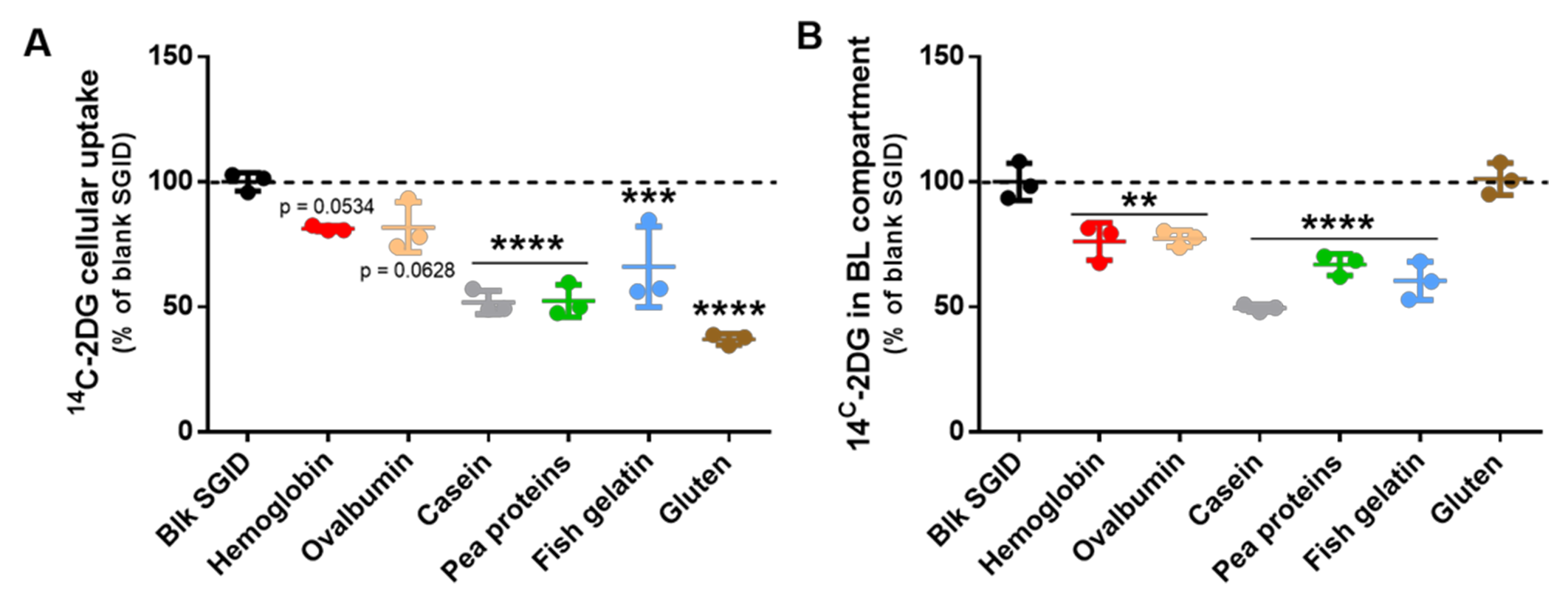

Supplement: Supplementary Figure 1 — Some digested dietary proteins decrease 2-DG intestinal transport in Caco-2/TC7 cells. After 1 h apical pre-incubation with 5 mg/mL digested proteins, Caco-2/TC7 cells differentiated on transwells were apically exposed for 8 min to the same digested proteins supplemented with 14C-2-deoxyglucose (2DG). Glucose uptake and absorption were then quantified by measuring radioactivity, respectively, in the cellular (A) and basolateral (B) compartments. Results are expressed as % of control (blk SGID) – mean ± SD (**, p < 0.01; ***, p < 0.001; and ****, p < 0.0001 compared to control – one-way ANOVA followed by Dunnett's test). [file Image_1.tif]

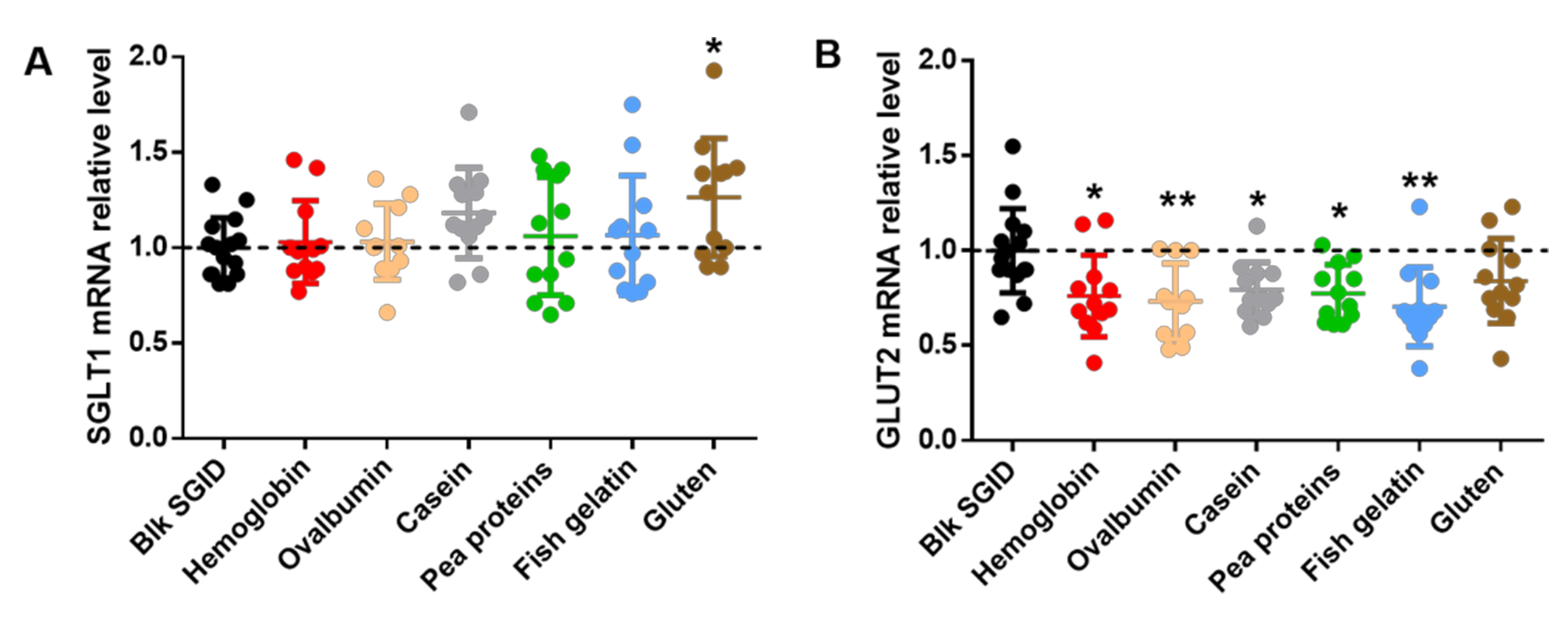

Supplement: Supplementary Figure 2 — Some digested dietary proteins decrease GLUT2 mRNA expression after 2 h incubation in Caco-2/HT29-MTX co-culture. SGLT1 (A) and GLUT2 (B) mRNA relative levels normalized to HPRT1 in Caco-2/HT29-MTX co-culture incubated for 2 h with 5 mg/ml digested proteins (hemoglobin, ovalbumin, casein, pea proteins, fish gelatin, and gluten). Control condition (Blk SGID) is set at 1. Mean ± SD (*, p < 0.05 and **, p < 0.01 compared to control – one-way ANOVA followed by Dunnett's test). [file Image_2.tif]
